# Oxysterols and Gastrointestinal Cancers Around the Clock

**DOI:** 10.3389/fendo.2019.00483

**Published:** 2019-07-17

**Authors:** Urša Kovač, Cene Skubic, Laura Bohinc, Damjana Rozman, Tadeja Režen

**Affiliations:** Centre for Functional Genomics and Bio-Chips, Institute of Biochemistry, Faculty of Medicine, University of Ljubljana, Ljubljana, Slovenia

**Keywords:** oxysterols, circadian rhythm, hepatocellular carcinoma, pancreatic cancer, colorectal cancer, ROR, LXR, FXR

## Abstract

This review focuses on the role of oxidized sterols in three major gastrointestinal cancers (hepatocellular carcinoma, pancreatic, and colon cancer) and how the circadian clock affects the carcinogenesis by regulating the lipid metabolism and beyond. While each field of research (cancer, oxysterols, and circadian clock) is well-studied within their specialty, little is known about the intertwining mechanisms and how these influence the disease etiology in each cancer type. Oxysterols are involved in pathology of these cancers, but final conclusions about their protective or damaging effects are elusive, since the effect depends on the type of oxysterol, concentration, and the cell type. Oxysterol concentrations, the expression of key regulators liver X receptors (LXR), farnesoid X receptor (FXR), and oxysterol-binding proteins (OSBP) family are modulated in tumors and plasma of cancer patients, exposing these proteins and selected oxysterols as new potential biomarkers and drug targets. Evidence about how cholesterol/oxysterol pathways are intertwined with circadian clock is building. Identified key contact points are different forms of retinoic acid receptor related orphan receptors (ROR) and LXRs. RORs and LXRs are both regulated by sterols/oxysterols and the circadian clock and in return also regulate the same pathways, representing a complex interplay between sterol metabolism and the clock. With this in mind, in addition to classical therapies to modulate cholesterol in gastrointestinal cancers, such as the statin therapy, the time is ripe also for therapies where time and duration of the drug application is taken as an important factor for successful therapies. The final goal is the personalized approach with chronotherapy for disease management and treatment in order to increase the positive drug effects.

## Introduction

### Cholesterol and Oxysterols

**Figure 1:**
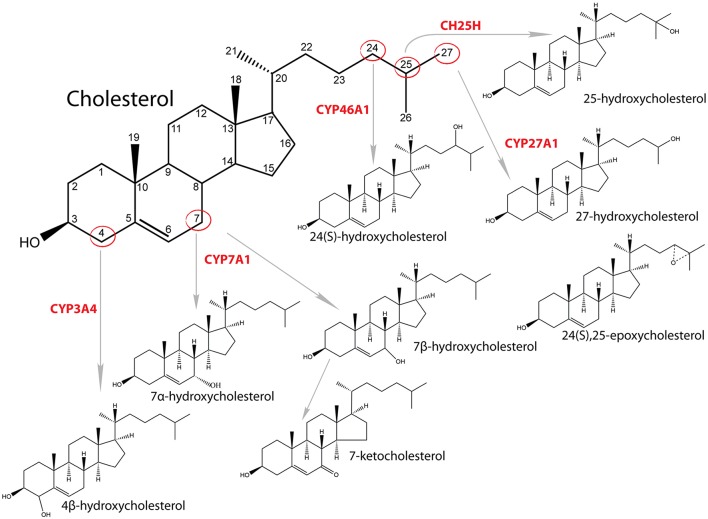
Synthesis of oxysterols and catalyzing enzymes.

Cholesterol is an essential molecule that participates in many cellular processes. It enables proper functioning of cellular membrane, is a precursor for synthesis of steroid hormones, oxysterols and bile acid, and functions as a signaling molecule regulating cell cycle, modifying proteins, and affecting its own synthesis (1–3). Most of the cholesterol in cells resides in the cellular membranes, where it plays a crucial role in stabilization of membranes, affects its fluidity and has an important role in lipid rafts (3). Oxysterols also have multiple functions, such as affecting membrane fluidity, regulating SREBP (sterol regulatory element binding transcription protein) signaling pathway through regulation of INSIGs (insulin induced genes) and by this sterol synthesis, interacting with NPC1 (NPC intracellular cholesterol transporter 1) and OSBP/OSBPL [oxysterol-binding proteins (like)] and, most importantly, are ligands and activators of several nuclear receptors, such as RORs (NR1F1-3, retinoic acid receptor related orphan receptors A, B, C), FXR (NR1H4, farnesoid X receptor alpha), PXR (NR1I2, pregnane X receptor), ESR1/2 (NR3A1/2, estrogen receptor alpha/beta), and LXR (NR1H3, liver X receptor alpha) (4, 5). The term oxysterol usually means oxidized sterols that are produced from cholesterol enzymatically or by auto oxidation (Figure 1). However, also other sterols, including intermediates of cholesterol synthesis can be oxidized at least enzymatically by cytochrome P450 (CYP) enzymes (6). Auto oxidation of cholesterol usually happens in the presence of reactive oxygen species and oxidation occurs on the B ring of sterol nucleus, mainly at positions C7 or C6. With this process 7α/β-hydroxycholesterol, 7-ketocholesterol and 6-hydroxycholesterol are formed (7, 8). Enzymatic synthesis includes the side chain oxidation by CYP or non-CYP. For example, 24- hydroxycholesterol is synthesized by CYP46A1, 25-hydroxycholesterol by 25-hydroxylase (non-heme iron-containing protein) and 27-hydroxycholesterol by CYP27A1 (7, 9). Other oxysterols are formed by oxidation of the sterol nucleus, like 4β-hydroxycholesterol synthesized by CYP3A4 or 7α-hydroxycholesterol by CYP7A1 (10). The concentration of oxysterols in normal healthy tissue and blood is 10^4^- to 10^6^-fold lower compared to cholesterol (11). In addition to endogenous synthesis, oxysterols can also derive from the diet. The cholesterol-rich food contains 10 to 100 μM concentration of oxysterols. Most common are 7α- and 7β-hydroxycholesterol, 7-ketocholesterol, cholestane-3ß,5α,6ß-triol, 5α,6α-epoxycholesterol, and 5β,6β-epoxycholesterol (12). When oxysterols were fed to humans, they were found in chylomicrons and lipoproteins (13).

SREBPs are transcription factors that activate transcription of genes necessary for cholesterol synthesis and uptake. Mammalian cells express three SREBP isoforms, SREBP-1a, SREBP-1c, and SREBP-2, which are responsible for expression of different lipid associated genes. SREBPs are located in endoplasmic reticulum (ER) together with SCAP (SREBP cleavage-activating protein) the escort protein, and INSIGs, the inhibitors of translocation. When cholesterol level declines, SREBPs are translocated to Golgi apparatus together with SCAP where they are proteolytic cleaved. A smaller SREBP is translocated to nucleus where it induces transcription of target genes (14). The major regulator of cholesterol homeostasis is SREBP-2 (15) as shown by the early mouse knockout experiments as well as in follow-up studies (16, 17). When SCAP is bound to INSIG, the vesicular transport of SREBP from ER is disabled. The cholesterol and oxysterols level regulate the INSIG inhibition of SCAP/SREBP transport (18, 19). Interestingly, cholesterol and oxysterols both induce the SCAP-INSIG interaction but by different mechanisms. Cholesterol acts by binding to SCAP and causing its conformational change that promotes binding to INSIG. Oxysterols, on the other hand, act directly on INSIG. For INSIG-2 it was shown that hydroxyl group on the side chain of sterols (22, 24, 25, or 27 position) is necessary for successful binding. A study confirmed these results, when 27-hydroxycholesterol levels were upregulated by CYP27A1 overexpression in mice or when primary hepatocytes were treated with 27-hydroxycholesterol, *Insig-2* expression was induced and SREBP-1 translocation was prevented (20). In this way, oxysterols regulate cholesterol synthesis and uptake through regulation of SREBP signaling pathway (18).

All these pathways are regulated by cholesterol and cholesterol synthesis intermediates but also by oxysterols themselves. Oxysterol synthesis itself is regulated in a similar manner as cholesterol and bile acid synthesis since many enzymes, transporters, and transcription factors are common. The excess of oxysterols is toxic for cells; therefore, oxysterols are transported to the liver, where they are metabolized to bile acid products and excreted from the body (21). FXR is a nuclear receptor of bile acids and is also the major regulator of bile acid synthesis. Hence, FXR regulates directly or indirectly expression and activity of oxysterol producing enzymes (CYP7A1, CYP27A1, CYP3A) (22). Also 22(R)-hydroxycholesterol has been shown to regulate expression of ABCB11 (ATP binding cassette subfamily B member 11, alternatively also BSEP- bile salt export protein) through FXR in hepatocytes (23). Since bile acids can act through different signaling pathways not connected to oxysterols, we will focus only on FXR direct involvement in selected cancers.

### Circadian Rhythm

Circadian clock as the inner rhythm in mammals is recognized as a cell-autonomous and self-sustaining mechanism, which controls almost every aspect of our life. The period of these rhythms is ~24 h long, thus circadian (from latin *circa*—approximately; *diem*—day) (24). The circadian rhythmicity is known to be a crucial endogenous process of organisms, described in almost every live species from cyanobacteria to human, and is capable to adapt to the environmental rhythm of the day. Circadian homeostasis in mammals is maintained by the central clock located in the suprachiasmatic nucleus (SCN) of the hypothalamus which orchestrates numerous clocks in peripheral tissues. The peripheral clocks have been observed in cells and tissues all over the mammalian body. Light, however, is not the only signal for entrainment of internal clocks. Systemic cues including hormones, body temperature, feeding/fasting cycles also influence the circadian rhythm in tissues throughout the body (25). The molecular basis of clock is constituted of periodical expression of clock genes driven by the autoregulatory transcription-translation feedback loops involving *cis*-regulatory elements such as E-boxes, D-boxes, and ROREs (ROR-elements) (26). The positive transcriptional loop is formed by transcriptional activators ARNTL/BMAL1 (Aryl Hydrocarbon Receptor Nuclear Translocator Like) and CLOCK (Clock Circadian Regulator, NPAS2 in neuronal tissue, Neuronal PAS Domain Protein). The CLOCK:BMAL1 heterodimer binds to conserved E-box sequences in target gene promotors of *PER1,2,3* (Period Circadian Regulator 1, 2, 3), *CRY1*,2 (Cryptochrome 1, 2), and *DEC1*,2 (Deleted In Esophageal Cancer 1, 2) genes contributing to the activation of their expression (27). PER and CRY proteins dimerize in the cytoplasm and after translocation to the nucleus inhibit further *CLOCK:BMAL1* transcription, forming the negative feedback loop. Also DEC1 and DEC2 can interact with the component of the SCN core clock genes. DEC1 and DEC2 proteins form dimers and translocate to the nucleus where they inhibit the activity of CLOCK:BMAL1 heterodimer (28). The degradation of PER, CRY and DEC proteins is essential for the restart of a new cycle of the transcription with ~24 h periodicity. An additional regulatory loop of BMAL1 and CLOCK dimer activity is through interaction with RORs and REVERBA (Nuclear Receptor Subfamily 1 Group D Member 1, NR1D1). RORs and REVERBA compete for ROR response element in the promotor region of *BMAL1*, where REVERBA acts as inhibitor and RORs as activators of *BMAL1* transcription. In turn, CLOCK:BMAL1 heterodimers activate *REVERBA* transcription. Besides E-boxes and ROREs, D-boxes play an important role as well. Transcription regulation through D-boxes goes via different transcription factors, such as DBP (albumin gene D-site binding protein) expressed in the SCN with a clear rhythm in the light-dark or constant dark conditions. The role of TIMELESS (timeless circadian regulator gene), which is mammalian ortholog of *Drosophila* TIM, remains ambiguous (29). In *Drosphila* TIM protein plays an important role and entrains the internal clock through light. After heterodimerization with PER, translocation to the nucleus occurs and transcription of core clock cycle genes is inhibited.

Regulation of circadian rhythm, however does not stop with transcription-translation regulatory loops. Post-translational changes such as phosphorylation, ubiquitination and acetylation as well as epigenetic changes have been proven to play an important role in regulation of circadian rhythms (30–32). PER1-3 proteins are subjects to temporal changes in phosphorylation by CKIε (casein kinase Iε) and CKIδ, reaching a peak right before its destruction. Their reversible phosphorylation is a dynamic process with kinases and phosphatases as counterparts, their net effect leading to altered PER2 protein stability and cellular location, ultimately affecting the length of circadian period (33). Additionally, CLOCK has been found to have HAT (histone acetyltransferase) activity (34). While HAT activity results in transcription chromatin states, its counterpart, histone deacetylase, would condense chromatin and silence gene expression. Indeed, SIRT1 (sirtuin 1), a NAD+ (nicotinamide adenine dinucleotide) dependent protein deacetylase, is a representative of HDAC (histone deacetylase) family, whose deacetylation is under circadian control. In the liver, SIRT1 deacetylates BMAL1 and PER2 as well as histone H3 in promotors of clock controlled genes (31, 35). With its reaction depending on NAD+, SIRT1 scopes far beyond its circadian regulation. Primarily known physiological effects of SIRT1 deacetylation were regulation of metabolism and response to oxidative stress. It therefore represents a functional link between cellular metabolic activity and stress response, cell proliferation and genome stability, standing at crossroads of the processes we are tackling (31, 36–38).

### Cholesterol and Oxysterols Around the Clock

**Figure 2:**
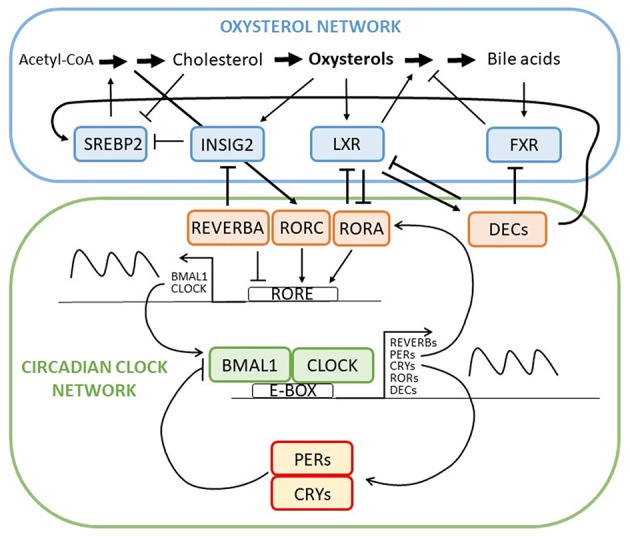
Intertwined oxysterol and circadian clock networks with multiple negative feedback loops and several contact points between oxysterol network and circadian clock regulation through RORA-LXR, REVERBA-INSIG, and DEC-FXR/LXR/SREBP interactions. Through contact points circadian network regulates circadian expression of oxysterol network genes and metabolites and *vice versa*.

Disorders of lipid metabolism are responsible for many adverse pathologies, including tumor development. It is already well-established that circadian clock controls every aspect of our life, also the lipid metabolism (Figure 2). The core circadian machinery is placed in the hypothalamus and orchestrates other peripheral clocks in tissues like liver, colon, and pancreas. Peripheral clocks can be synchronized trough different channels. They can be directly synchronized by SCN (neural and hormones signals), trough food entrainment or body temperature (39). Lipid metabolism focusing on cholesterol metabolism is under a daytime specific regulation. Recently it was shown that adropin, identified as a protein that has a role in maintaining the energy homeostasis, could represent a link between circadian clock and cholesterol metabolism. Expression of adropin is diminished by cholesterol and 7-oxygenated sterols that can modulate the RORA/C signaling. The RORA/C and REVERB receptors might thus link adropin and its synthesis to circadian rhythm of lipid metabolism which is a novel and important connection (40).

Liver X receptors whose natural ligands are oxysterols have been found to regulate *Dec1*, a transcription factor involved in hepatic clock system and metabolism (41). *In vivo* observation showed reciprocal suppression between RORA and LXR (42). Research on *Reverb*α mutant mice showed a circadian controlled expression of *Cyp7a1*. Furthermore, Martelot et al. also demonstrated that REVERBA cooperates in modulation of cholesterol and bile acid synthesis through control of SREBP. Thus, REVERBA control the rhythmic abundance of *Insig2* and is further responsible for diurnal translocation of SREBP to the nucleus. The circadian activation of SREBP drives the circadian transcription of cholesterol biosynthesis genes and hence the oxysterols, which activate LXR. The cyclically activated LXR is responsible for cyclic expression of CYP7A1 (43). Both, *Cyp7a1* and *Cyp27a1* showed circadian expression where sex differences in circadian variation were observed, indicting the importance of sex when planning the therapy (44). In the *Clock* mutant mice on a diet containing cholesterol and cholic acid showed reduced and disrupted *Cyp7a1* expression and high liver cholesterol accumulation (45). Timed high fat diet in mice, on the other hand, resulted in changed *Cyp7a1* expression via PPARα (peroxisome proliferator activated receptor alpha) as well as increased hepatic cholesterol (46). *Cyp7a1* anomalies were found in *Reverb*α deficient mice as well as *Per1*^−/−^ and *Per2*^−/−^ mice, where bile acid homeostasis was disrupted (47, 48). Other CYP enzymes involved in cholesterol biosynthesis have been reported in association with circadian clock metabolism. CYP3A has a circadian pattern, but researched did not find it to be important for drug therapy due to small changes observed (49). mRNA levels and metabolic activity of CYP3A4 in serum-shocked HepG2 cells showed a 24 h rhythmicity. DBP was responsible for CYP3A4 activation (50). CYP8B1, an important player in bile acid and liver metabolism, is also under the RORA regulation resulting in its diurnal rhythm and fasting induction (51). Furthermore, DEC2 was shown as an important regulator that convey the circadian signal to the liver that has role in *CYP7A, CYP8B*, and *CYP51A1* expression (52).

## Hepatocellular Carcinoma (HCC)

Hepatocellular carcinoma (HCC) is one of the most common causes of death worldwide and represents the most common primary malignancy in the liver. It is commonly associated with chronic hepatitis B virus (HBV) or hepatitis C virus (HCV) infection, aflatoxins and alcoholic cirrhosis (53). It is a global health problem and over 80% of HCC cases are seen in developing countries with the highest incidence in areas endemic for HCV and HBV (54). Most patients with HCC have liver cirrhosis but there is also a number of cases where patients with non-alcoholic fatty liver disease (NAFLD) and without cirrhosis develop HCC, demonstrating the role of NAFLD in its pathogenesis (55, 56). The incidence is increasing rapidly and is higher in men compared to women (57). Other types of liver cancers like intrahepatic cholangiocarcinoma, hepatoblastoma, and angiosarcoma are not so common in comparison to HCC. The molecular pathogenesis of HCC is extremely complex where circadian clock and lipid metabolism play an important role (58). Genetic and epigenetic alterations have been shown to also drive hepatocarcinogenesis. Knowing the crucial signaling pathways in HCC will enable us to provide better therapies to treat this cancer (59). The overview of factors associated with HCC are presented in Table 1.

**Table 1:** Oxysterol-circadian factors influencing HCC development.

**Factor**	**Notes**	**Proposed function**	**References**
Oxysterols	Increased in serum of NAFLD, HCV infected patients	Potential biomarkers	(60, 61)
	Treatment of HepG2	Cytotoxicity	(62)
25-hydrohycholesterol	Treatment of rat hepatoma cells	Pro-apoptotic	(62, 63)
25-hydroxycholesterol and OSBPL8	Treatment and overexpression in HepG2	Pro-apoptotic	
OSBPL8	Downregulation in Huh7 and HepG2	Pro-proliferative	(62)
	Overexpression in HCC cell line	Pro-apoptotic	(64)
LXR	Lower expression in HCC tumors	Lower post-operative survival rate	(65)
	Activity	Anti-proliferative via SOCS3	(66)
FXR	Lower expression and activity in HCC tumors	Association with multiple malignant characteristics	(67–72)
	Overexpression in HCC cell lines	Anti-proliferative, suppressed tumor growth in nude mice	(69)
	Downregulation in HCC cell lines	Pro-proliferative, increased migration and invasion, accelerated tumor growth in nude mice	(70)
SREBP1	Increased expression in HCC cell line	Required for tumor proliferation	(73)
CLOCK (NPAS2)	Increased expression in HCC tumors		(74)
	SNPs	Associated with increased risk for HCC, survival of TACE treated patients	(75)
PER3	SNP	Association with survival	(76)
PER2	Knockout mice	Higher diethylnitrosamine induced carcinogenesis	(77)
Chronic circadian disruption	Spontaneous hepatocarcinogenesis in mice	Metabolic disruption, CAR activation	(77–79)
TIMELESS	HepG2	Oncogenic	(80)

In patients infected with HCV, the cholesterol transport in the liver is modified and serum concentration of cholesterol was lower compared to healthy individuals. Interestingly, the serum levels of oxysterols 4β-hydroxycholesterol, 25-hydroxycholesterol, and 7α-hydroxycholesterol were elevated despite lower cholesterol concentration. When patients with HCV were treated with anti-viral therapy the oxysterols where reduced back to normal values. Further analyses indicated that these oxysterols were probably not elevated because of the activity of liver enzymes CYP3A4 and CYP7A1. *CYP7A1* expression was unchanged in patients with HCV (61) and CYP3A4 activity was even downregulated (81). Direct effect of higher oxysterol concentrations on the development of HCC remains unknown, but oxysterols may be potential biomarkers and even potential novel targets for inhibiting disease progression (61). One of the major primary risk factors for HCC is NAFLD. With increasing control over hepatitis infections, it is predicted that NAFLD will soon become the major risk factor for HCC development. NAFLD is a complex multi systemic disease, caused by many factors such as genetics, dietary, environmental, and others (82, 83). In NAFLD patients, serum cholesterol is elevated and consequently some oxysterols (4β-, 25-, and 27-hydroxycholesterol) are also elevated. These oxysterols can affect the resorption of cholesterol in some tissues, through LXR activation, which regulates ATP-binding cassette transporters. In this way oxysterols affect the cholesterol concentration in NAFLD, which can evolve in HCC (60). In rat model with induced hepatoma tumor in the liver, the measurements showed elevated levels of following oxysterols: 24S-, 25- and 27-hydroxycholesterol, and 24S,25-epoxycholesterol, which are all known to bind to LXR. In these tumors, the ABCA1 (ATP binding cassette subfamily A member 1) and ABCG1 (ATP binding cassette subfamily G member 1) cholesterol transporters were elevated on mRNA and protein levels, most likely through LXR activation. There is some data indicating that 25-hydroxycholesterol has a pro-apoptotic function in rat hepatoma cells and is able to induce sub-Gl apoptosis (63). Authors suggested that local addition of 25-hydroxycholesterol in combination with other drugs could be potentially used in treatment of hepatoma patients. HCC cells have changed metabolism to sustain the fast proliferation. Because of this, many cellular functions are altered, including cholesterol metabolism and transport. It was shown that oxysterols are cytotoxic, can induce cell death in HepG2 cells, and can suppress the growth of HCC cells by inhibiting ACAT2 (Acyl-coenzyme A:cholesterol acyltransferase) enzyme, which results in intracellular unesterified oxysterol accumulation (62). When OSBPL8 (also known as ORP8) is downregulated, cell proliferation is promoted. When ORP8 was overexpressed in HepG2 cells and 25-hydroxycholesterol was added, the ER stress and apoptosis were promoted (62). ORP8 overexpression was already enough to trigger apoptosis in primary HCC cells isolated from human liver and cell lines (64). Lower LXR expression was associated with lower post-operative survival rate (65). LXR regulated HCC response to TGFβ1 (transforming growth factor beta 1), which acts as cytostatic and pro-apoptotic (84). LXR suppressed HCC proliferation through activation of SOCS3 (suppressor of cytokine signaling 3) (66).

FXR's role in HCC development was indicated when whole body *Fxr* knockout mice developed spontaneous HCC by the age of 14 months (85, 86). These mice had abnormal level of bile acids in the serum and administration of cholestyramine decreased HCC incidence (85). Many studies confirmed a significant decrease in FXR expression and activity in human HCC tumors and this was associated with multiple malignant characteristics (67–72). However, intestine-specific *Fxr* reactivation in *Fxr* knockout mice restored bile acid enterohepatic circulation, limiting hepatic inflammation and proliferation while preventing spontaneous hepatocarcinogenesis (87). Also only 20 and 5% incidence of hepatic tumors was observed in hepatocyte specific and enterocyte specific *Fxr* knockout mice, respectively. No serum or hepatic increase in bile acid level was observed in either of cell specific knockout models and no change in expression of cell-cycle regulators (88). These results lead to a hypothesis that high bile acid levels and FXR deregulation are needed for HCC development. There is, however, a mounting data pointing to a direct involvement of FXR in tumor suppression.

Overexpression of FXR decreased proliferation of HCC cell line and suppressed tumor growth in nude mice (69). Inhibition of FXR expression resulted in enhanced cell proliferation, migration and invasion in HCC cell lines and accelerated tumor growth in nude mice. FXR was shown to directly interact with β-catenin and repress its transcriptional activity (70). FXR also directly upregulates NDRG2 (N-myc downregulated gene family member 2) (72). NDRG2 is a known tumor suppressor, whose expression is reduced in HCC samples and correlates with aggressive tumor characteristics (72, 89). FXR also directly inhibited gankyrin expression via C/EBPβ-HDAC1 (CCAAT enhancer binding protein beta/ histone deacetylase 1) complex (90). Gankyrin is a known oncogene and is increased in HCC (91). In this study, they also showed that long-lived Little mice, which have high FXR liver expression, do not develop liver cancer after diethylnitrosoamine injection in comparison to wild type mice (90). FXR upregulated mir-122 which suppressed proliferation of HCC cells and the growth of HCC xenografts *in vivo* (71). In HCC human tumors miR-122 was significantly downregulated and this correlated with FXR expression (71). FXR and its agonist waltonitone repressed HCC cell proliferation by activating mir-22 repression of CCNA2 (Cyclin A2) (92). In HCC tumors FXR and miR-22 were downregulated while CCNA2 expression was opposite (92, 93). FXR also repressed inflammation via SOCS3 and via inhibiting NFκB (nuclear factor Kappa B) signaling (94, 95). FXR is a direct regulator of SOCS3, which enabled FXR-mediated cell growth repression, and their expression is correlated in HCC human samples (96). In summary, a direct role of FXR in protection against hepatocarcinogenesis could also be *via* defense against bile acid-induced injury, prevention of liver injury and apoptosis, prevention of ROS generation, promoting liver repair and generation after injury, suppressing cancer cell proliferation, etc. (97). There are some potential explanations available for the mechanisms behind FXR downregulation in HCC. In HCC cell lines miR-421 downregulated FXR and by this promoted cell proliferation, migration and invasion (98). Increased expression of miR-421 and its correlation with patient's survival was confirmed in HCC patients (99). Another factor decreasing the expression and activity of FXR is inflammation and inflammatory cytokines were shown to inhibit HNF1A (HNF1 homeobox A) binding to *FXR* promotor (68). SIRT1 overexpression leads to deacetylation of FXR in the mouse liver, and inverse expression of SIRT1 and FXR was confirmed in human HCC samples (100). In spite of many tumor suppressor actions discovered, there is also some caution needed. FXR also upregulates FGF19 (fibroblast growth factor 19) which is anti-cholestatic and anti-fibrotic factor in the liver and was proposed for the treatment of NASH. However, FGF19, but not its rodent counterpart FGF15, is potentially pro-tumorigenic as shown by ectopic expression in mice (101). FGF19 expression is upregulated in HCC samples and correlates with poor prognosis (102). This is an important factor to consider when translating results from studies in rodent models to humans. However, new engineered FGF19 variants have been developed excluding pro-tumorigenic activity and are currently in evaluation for the treatment of NASH (103).

More and more results show the importance of SREBPs, especially SREBP-1, as a link between oncogenic signaling and tumor metabolism. It was shown that cancerous cells have higher *de novo* lipogenesis, which is required for rapid tumor proliferation. In the cancer cells, SREBP-1 is highly activated and it was shown, that pharmacological targeting of SREBP-1 greatly inhibits tumor growth. This rises hope for SREBP-1 to be potential anti-cancerous target (73). Higher expression of SREBP-1 was shown in different cancer cell lines (104) and higher expression of SREBP-1 is associated with many metabolic diseases; including NAFLD and NASH. One of the factors activating SREBP-1 in HCC is hepatoma-derived growth factor (HDGF). Co-expression of HDGF and SREBP-1 is an indicator of poor HCC prognosis. HDGR knockdown or its mutation in HepG2 cells resulted in decreased expression of SREBP-1 targeted genes (105). A study on a mouse model of HCC showed that lipid biosynthesis (fatty acids and cholesterol) is crucial for HCC development and that targeting SREBP pathway can be used as an anti-tumor strategy (106). Study on hepatic stellate cells, which have an important role in liver fibrosis, showed that in these cell line INSIG-1 and INSIG-2 expression is downregulated. By downregulation of INSIGs, SREPB signaling is promoted, which is the characteristic of HCC and crucial for cancer proliferation (107). Bioinformatics analysis showed that two miRNA (has-miR-221 and has-miR-29c) are potential HCC diagnostic markers and that they have one common target, which is *INSIG1* (108). All these data show the importance of SREBP signaling and its potential as drug target.

Data on human and mice reported a connection between circadian rhythms and liver disease where alterations in rhythmicity had a profound effect on metabolic pathways resulting also in adverse outcomes, like HCC. NPAS2 or CLOCK was shown to have a critical role in HCC and expression of the protein was significantly upregulated in HCC patients (74). In other study, single nucleotide polymorphisms (SNPs) in *NPAS2* were associated with the increased risk for HCC or with overall survival in HCC patients treated with transcatheter arterial chemoembolization (TACE) (75). The rs2640908 polymorphism within *PER3* was also associated with overall survival in HCC patients treated with TACE (76). SNPs from circadian negative feedback loop genes were further studied and were suggested to have an independent role as prognostic biomarkers for prediction of HCC clinical outcome (109). Study on mice showed that circadian disruption due to *Per2* mutation or jet lag has a profound role in liver carcinogenesis induced by diethhylnitrosamine (77). Other research addressed the importance of the circadian clock metabolism in hepatocarcinogenesis where the changes in expression of several core clock genes were downregulated (*BMAL1, CLOCK, PER1, PER2, CRY1, CRY2*), while upregulation of clock related genes (*NR1D1* and *DBP*) was observed (110). Circadian metabolism and carcinogenesis are highly interconnected and the connection is complicated. Kettner et al. demonstrated that chronic circadian disruption induces NAFLD and spontaneous hepatocarcinogenesis, promotes genome-wide deregulation and metabolic disruption, and activates CAR (NR1I3, constitutive androstane receptor) which drives NAFLD to NASH, fibrosis and HCC progression (78). Chronic circadian disruption in mice had a profound effect on metabolism, what was also observed in humans and circadian disruption with or without steroid receptor Coactivator-2 (SRC-2) ablation showed association with human HCC gene signature (79). The TIMELESS protein has oncogenic function in human HCC as well (80). Studies on HepG2 cell line demonstrated the importance of *RORA* in different steps in liver carcinogenesis (111).

## Pancreatic Cancer (PC)

Pancreatic cancer is one of the most malignant cancers, estimated to cause 432 242 deaths in 2018 and ranking seventh among estimated cancer deaths in the world (112). Estimated 5-year survival rate is extremely low and only 4% of patients will live 5 years after diagnosis. An important risk factor is smoking, since around 20% of tumors are attributable to smoking. Other risk factors include family history of chronic pancreatitis or pancreatic cancer, male sex, advancing age, diabetes mellitus, obesity and occupational exposure (113, 114). Novel epidemiological studies propose that dietary factors also impact pancreatic cancer risk. In a large population saturated fatty acid intake was linked to higher pancreatic cancer risk, while omega-3 fatty acids, increased vitamin C and vitamin E intake lowered cancer risk (115). Pancreatic cancer patients have unspecific signs, making the diagnosis difficult, often discovering already advanced stages of the disease. The overview of factors associated with PC are presented in Table 2.

**Table 2:** Oxysterol-circadian factors influencing PC development.

**Factor**	**Notes**	**Proposed function**	**References**
Cholesterol, 5α,6α-epoxide, and lanosterol	Increased in serum of PC patients	Potential biomarkers	(116)
OSBPL5	Increased expression in PC tumors	Higher invasion and poor prognosis	(117, 118)
OSBPL3	Increased expression in PDAC tumors	Poor prognosis	(119)
LXR	Activation by ligands in PDAC cell lines	Anti-proliferative	(120)
FXR	Increased expression in PC tumors	Poor prognosis	(121, 122)
	Increased expression in PC tumors	Better prognosis	(123)
	Inhibition in PC cell lines	Anti-proliferative, lower migration and invasion	(121, 122)
	Increased expression in PC cell lines	Regulator of FAK/JUN/MUC4 pathway	(124)
BMAL1	Lower expression in PC tumors	Poor prognosis	(125, 126)
	Knockdown in PC cell lines	Anti-apoptotic, pro-proliferative	(126)
SIRT1	Significant down regulation in PC tumors	Lower mortality rate	(127)

Very little is known about the oxysterol role in the development of pancreatic cancer. Serum cholesterol, 5α,6α-epoxide, and lanosterol were identified as highly discriminating between pancreatic cancer patients and healthy subjects (116). Two meta-analyses showed that dietary cholesterol may be associated with pancreatic cancer in worldwide populations (128, 129). OSBPL have been shown to be involved in pathology of PC. Increased expression of OSBPL5 was associated with higher invasion and poor prognosis of pancreatic cancer patients (117, 118). In pancreatic ductal adenocarcinoma (PDAC) patients, OSBPL3 was found as differentially expressed in four studies and was correlated with poor prognosis of these patients (119). The role of nuclear receptors also remains unknown. Activation of LXR by synthetic ligands had anti-proliferative effects on PDAC cells (120). Very few studies measured the expression FXR and its role in pancreatic cancer. Moreover, there are conflicting results available. The increase in FXR mRNA and protein expression was confirmed in pancreatic tumors in comparison to adjacent tissue, and high FXR expression was correlated with the poor prognosis. FXR inhibition reduced cell proliferation, migration and invasion in pancreatic cancer cell lines (121, 122). It was proposed that FXR induced phosphorylation of SP1 (Sp1 transcription factor) and by this promoted cancer progression (122). While another study found correlation between high FXR expression with less aggressive phenotype, smaller tumors, absence of metastases and better prognosis (123). The difference in FXR expression and correlation between studies can simply be attributed to a low sample number, different groups of patients, different length of time in survival analyses, correlation with other factors etc. However, bile acids were increased in plasma and pancreatic juice in PC patients, and the mouse model confirmed the correlation between the increase in serum bile acids level with the severity of the disease (124). FXR was significantly increased in pancreatic cancer cell lines in comparison to normal pancreatic cells and FXR was found as the regulator of FAK (PTK2, protein tyrosine kinase 2)/c-Jun (JUN, Jun proto-oncogene, AP-1 transcription factor subunit) / MUC4 (mucin 4) signaling pathway (124). Similar to HCC, SREBP1 pathway and *de novo* lipogenesis is upregulated in PC. Study on 60 PC patients showed high correlation between elevated SREBP1 expression and poor disease prognosis. Inhibition of SREBP1 in a mouse model lead to decreased *in vivo* weight of tumor, indication the importance of SREBP1 upregulation for cancer cell growth (130). Study on PC cell model showed that inhibitors of SREBP1 decrease PC cell viability and proliferation. This indicate that targeting SREBP1 pathway is potential target for PC disease management and should be further explored (131). One of the potential molecules for targeting SREBP1 is resveratrol. Recent study on mouse model of PC and human cell lines treated with gemcitabine (chemotherapy medication) showed that resveratrol suppressed stemness induced by gemcitabine. Resveratrol should be considered as an additive for chemotherapeutic drugs (132).

Circadian activity in pancreas is also controlled by SCN (133, 134). The clock genes expression (*BMAL1, PER1, PER2, PER3, CRY2, TIMELESS, and CK1*ε) is altered in PDAC which is the most common type of pancreatic malignant tumors (135, 136). In humans, lower *BMAL1* expression was associated with poorer survival rate, and correlated with higher tumor stage, poorer histological differentiation, and increased vascular invasion in PDAC (125, 126). Since knockdown of *Bmal1* resulted in an anti-apoptotic and pro-proliferative profile, while its overexpression had the opposite effect, *Bmal1* can act as an anti-oncogene by binding directly to *p53* promoter region (126). Since circadian rhythms and metabolic pathways go both ways, and pancreatic cancer risk is increased in people with metabolic syndrome (137), the role of metabolism and its disruption plays an important role in pancreatic cancerogenesis. A recent study evaluating dietary influence on cancer risk in women confirmed the role of obesity (138). *Bmal1* and its downstream regulation seems to play a crucial role in pancreatic regulation of metabolic processes, as *Bmal1* knock out in mouse β-cells led to glucose intolerance and development of diabetes (139). Since glucose metabolism is also under the circadian control, it is not surprising that alterations in clock genes (*Clock, Bmal1*) lead to impaired glucose tolerance (140), with a profound role in pathogenesis of pancreatic cancer (141). Deregulation of SIRT1, which regulates the central and peripheral clock, has an important role in PDAC as well (127).

## Colorectal Cancer (CRC)

Colorectal cancer is the third most commonly diagnosed cancer, the second most common cause of oncological death in the world estimated in 2018 (112) and as such represents a major public health issue worldwide. About 6% of CRCs are predetermined by a defined hereditary syndrome, while around 25% of CRCs are familial, the latter being tightly intertwined with many of the modifiable risk factors as well as genetic factors. Non-modifiable risk factors for CRCs include increasing age, male gender, African American race and residence in high-income countries (142), while modifiable factors include obesity, moderate to heavy alcohol consumption, smoking, high consumption of red or processed meat and a diet with low fiber, fruit, and vegetables intake (112, 142). Also, genetics plays an important role in its pathogenesis (142). In CRC one of the most commonly dysregulated pathway is the Wnt/β-catenin signaling, leading to β-catenin accumulation, ultimately resulting in activation of several gene targets, including oncogenes that contribute to the development of cancerous phenotype (143). Its component APC (APC regulator of WNT signaling pathway) mutations are present in majority of sporadic CRCs (144). Since onset and progression of CRC are tightly intertwined with its mutational pathways, detailed knowledge of their regulation is crucial for obtaining new markers in order to better understand risk factors as well as treatment and prognosis. The overview of factors associated with CRC are presented in Table 3.

**Table 3:** Oxysterol-circadian factors influencing CRC development.

**Factor**	**Notes**	**Proposed function**	**References**
Total serum cholesterol	Increased in humans	Higher risk for CRC	(145, 146)
CYP7A1	SNP and haplotype	Associated with CRC	(147–150)
Oxyphytosterols	Treatment of CRC cell lines	Anti-proliferative, pro-apoptotic	(151)
Dietary oxysterols	Treatment of Caco-2	Pro-apoptotic	(152)
27-hydroxy cholesterol	Treatment of CRC cell lines	Anti-proliferative	(153)
7α- and 7β-hydroxycholesterol, 5α,6α-epoxycholesterol, and 7β-hydroxysitosterol	Treatment of Caco-2	Pro-apoptotic	(154–156)
7β-hydroxycholesterol	Treatment of Caco-2	Induced expression of inflammatory and chemotactic cytokines	(157)
7-ketocholesterol	Treatment of Caco-2	Reduced barrier functions, anti-apoptotic, induced viability, lower inflammatory response	(158, 159)
	Treatment of HT-29	Induction of ER stress	(160)
	Treatment of Caco-2	Mitochondrial functionality	(161)
25-hydroxycholesterol	Treatment of Caco-2	Reduced barrier functions, anti-apoptotic, and induced viability	(158)
	Treatment of DLD-1	Induction of cell death, anoikis	(162)
	Treatment of Caco-2	Enhanced IL1B induction of IL8	(163)
LXR	Induction by agonist in CRC cell lines	Anti-proliferative	(164)
CYP8B1, CYP46A1, CYP2R1	Higher expression in CRC	Poor prognosis	(165)
CYP7B1	Higher expression in CRC	Good prognosis	(165)
OSBPL9	Downregulation in CRC tumors	Poor prognosis	(166)
LXR	Increased expression	Good prognosis	(167)
	Activation in HT-92	Anti-proliferative	(168, 169)
FXR	Decreased expression in CRC tumors	Poor prognosis	(170–175)
	Knockout in mice	Increased susceptibility to chemically-induced	(171, 176)
	Overexpression in CRC cell lines	Reduced tumor growth, anti-proliferative, pro-apoptotic	(92, 171, 175–177)
PER1, PER3	Decreased expression in CRC tumors	Poor prognosis	(178)
PER1, BMAL1	Decreased PER1 and increased BMAL1 in CRC tumors	Poor prognosis	(179)
PER2	Increased in CRC tumors	Good prognosis	(178)
RORA	SNPs	Risk of development of CRC	(180)
	Lower expression in CRC tumors	Good prognosis	(180)
SIRT1	Transgenic mice and human tumor specimens	Suppression of *in vivo* tumor formation	(181)

High total cholesterol level is associated with a higher risk for colon cancer in men (145). Another study taking into an account also genetic factors again confirmed a link between hypercholesterolemia and colorectal cancer risk (146). A *CYP7A1* haplotype and SNPs were significantly associated with colorectal cancer and it was proposed to be the effect of fecal bile acids (147–150). Whole grain food significantly reduces colon cancer risk and phytochemicals that are involved in this protection are especially interesting (182).

Studies in colon cancer cell lines indicate that different oxysterols are cytotoxic and induce apoptosis. A study of wheat bran showed that it contains several different oxyphytosterols with anti-proliferative effect on colon cancer cell lines, some could also induce apoptosis (151). Dietary-representative oxysterol mixture induced apoptosis in differentiated colonic CaCo-2 cells (152). A study in major cell line models for CRC, Caco2 and SW620 cells, showed that 27-hydroxycholesterol decreased proliferation of these cells (153). This effect was not due to cellular cytotoxicity or induction of apoptosis and it was independent of nuclear receptors LXR and ESRs. 7α- and 7β-hydroxycholesterol, 5α,6α-epoxycholesterol and 7β-hydroxysitosterol were all able to induce apoptosis in human colon cancer cell line Caco-2 (154, 155). 7β- hydroxycholesterol was cytotoxic to colon cancer cell lines in concentrations from 3 to 10 μM (156). 7-ketocholesterol and 25-hydroxycholesterol reduced barrier functions, apoptosis and induced viability of Caco-2 cells (158). 7-ketocholesterol besides decreasing epithelial barrier also induced inappropriate development of inflammatory response to food (159). 7-ketocholesterol induced ER stress in HT-29 colon cancer cell line (160) and affected mitochondrial functionality in Caco-2 cells, while co-treatment with 7-ketostigmasterol reduced the toxic effect (161). Only 25-hydroxycholesterol, but not the 22-(R)-hydroxycholesterol and other oxysterols (7 beta-hydroxycholesterol and 5-cholesten-3beta-ol-7-one) induced anoikis, a type of programmed cell death, in DLD-1 cells (162). Some of these effects could be through activation of LXRs, since LXR induction by GW3965 had anti-proliferative effects on colon cancer cells (164).

On the other hand, oxysterols also affect expression of inflammatory molecules in colon cancer cells. Inflammatory bowel disease is an important risk factor for development of CRC and chronic inflammation and oxidative stress are part of pathogenesis (183). 7β-hydroxycholesterol induced expression of key inflammatory and chemotactic cytokines in CaCo-2 cell line (157). 25-hydroxycholesterol pre-treatment enhanced IL1B (interleukin 1 beta) induced IL8 (interleukin 8) production in Caco-2 cells (184). Representative mixture of oxysterols increased oxidative stress in differentiated Caco-2 cells and was followed by the production of cytokines IL6 (interleukin 6) and IL8 (163). In CRC patients, serum level of IL8 was increasing with the progression of cancer (185). Expression of enzymes and oxysterol receptors is modulated in CRC. Human tissue microarray analyses revealed significantly higher protein expression of CYP2R1, CYP7B1, CYP8B1, CYP46A1, CYP51A1, while CYP27A1 and CYP39A1 had no significant change, in primary colorectal tumor compared to normal colonic mucosa (165). In this study, they also showed a significant association between patient prognosis and survival with CYP expression. For example, higher expression of CYP8B1, CYP2R1, CYP27A1, and CYP46A1 in tumor was associated with poor prognosis, while high CYP7B1 expression correlated with good prognosis. OSBPL1A short transcript variant was down-regulated in colon cancer tumors, but the long variant was unchanged in comparison to normal samples (186). OSBPL9 was included in a gene expression signature as a predictor of survival in colon cancer and was downregulated in poor prognosis patients (166).

LXR expression was proposed to be a prognostic indicator for CRC and its expression was associated with favorable clinical outcome. Positive LXR expression was associated with better survival rate and there was a significant negative association between LXR expression and vascular invasion, but no association was found between LXR expression and the patient's age, sex, tumor size, grade or TNM (tumor/node/metastasis) stage (167). A genome wide analyses revealed regulatory programs of LXR activation which lead to inhibition of HT29, a colorectal cancer cell line, proliferation (168). LXR was shown to directly bind to β-catenin and suppress its activity and cellular proliferation (169). A recent review has already summarized FXR and bile acids role in the development of colon cancer (187). Several studies showed that FXR expression is reduced in intestinal tumors in humans and that it is inversely correlated with the degree of malignancy and clinical outcome (170–175). The FXR knockout mice have increased susceptibility to chemically-induced colorectal carcinogenesis, while FXR overexpression in gut cells reduced tumor development and growth (171, 176). The activation of FXR was shown to suppress proliferation and induce apoptosis in colon cancer cell lines (171, 177). The observed downregulation of FXR expression is due to increased methylation of *FXR* promotor by the loss of APC function, which was confirmed in CRC cell lines, animal models and colonic tumors from patients (174, 188, 189). However, *FXR* is also downregulated by intestinal inflammation, western diet, and microRNA (187, 190). The current hypothesis is that the decreased FXR regulation in combination with Western diet and hence higher levels of secondary bile acids results in pro-tumorigenic colon environment leading to the development of colon cancer. Moreover, recent studies showed that FXR exhibits anti-cancerogenesis effects beyond regulation of bile acid level, by also affecting other cellular signaling pathways in colon cells. A study in mouse and organoid models, showed that FXR regulated proliferation of intestinal cancer stem cells (191). FXR repressed proliferation of colon cells by inhibiting the *MMP7* (matrix metallopeptidase 7), a known intestinal tumor promotor expression; by activating mir-22 repression of CCNA2 (Cyclin A2); and by activating the EGFR/SRC (epidermal growth factor receptor/SRC proto-oncogene) pathway in colon cells (92, 175, 177). Upregulation of SREBP1 pathway is also present in CRC (192). Inhibition of SREBP1 in CRC shows promising result for cancer management. It was shown that ordonin (diterpenoid isolated from *Rabdosia rubescens*) reduced expression of *SREBP1* and induce apoptosis in CRC cells cultures (193).

Epidemiological studies have shed light on connection between circadian disruption and elevated risk of cancer, including colorectal cancer (128, 194, 195). Meta-analysis showed increased incidence of colorectal cancer in people with long-term exposure to night light (shift work). The proposed mechanism for circadian disruption on oncogenesis is melatonin suppression and loss of its protective effect against cancer via apoptosis, anti-angiogenesis, anti-oxidation and regulation of the immune system. One of the proposed additional factors is low level of 25-hydroxyvitamin D due to lower sun exposure (128). In mouse models, physical destruction of the SCN as well as functional disturbance of circadian rhythms (chronic jet lag) resulted in accelerated tumor growth in transplantable tumor models (Glasgow osteosarcoma and Pancreatic adenocarcinoma), indicating a role of circadian system in controlling malignant growth (196). In human CRC, there are multiple studies reporting abnormal expression of circadian genes including altered expressions of *CLOCK, BMAL1, PER1, PER2, PER3*, and *CK1*ε (179, 197–200). Whether the disruption originates in core clock genes and drives tumorous transformation or measured disruption is a consequence of cancer, remains unclear. However, studies suggest that clock genes have an important role in tumor suppression (201). Although clinical correlations between specific mechanisms of clock gene disruption and colorectal cancer phenotype and prognosis have not been conclusive, some pathways show typical clinical and pathological features. Decreased expression of *PER1* and *PER3* in tumor tissue as such indicate poorer survival rate (178), decreased *PER1* and high *BMAL1* expression correlate with poorer outcome and liver metastasis (179), and high *PER2* expression correlate with significantly better disease outcome (178). In recent systematic evaluation of genetic variants in the circadian pathways connected with CRC, examining 119 SNPs in *RORA* was proposed as potentially important marker for CRC risk and prognosis. While people carrying SNP in *RORA* were much more inclined to developing CRC, lower RORA correlated with better differentiated tumors and better disease outcome (180). On the other hand, changes in cellular metabolism may cause circadian disruption, further influencing colorectal cancer phenotype (202). It has been shown that SIRT1 suppresses colorectal tumor formation *in vivo* by β-catenin deacetylation (181). A recent study showed that both metabolic and circadian dysregulation progressed during cancer progression. Their findings suggested that clock-related glycolysis genes alterations might add to a clock-driven rewiring of metabolism, connected to cancer progression and altering response to cancer therapy (203).

## New Therapeutic Strategies

### Targeting Oxysterol-Cholesterol Network

Statins are widely used lipid-lowering drugs which inhibit HMGCR, a rate limiting enzyme from cholesterol synthesis. Statins have been proposed in several studies as potential drugs used for reducing the risk of development and mortality in gastrointestinal cancers. Statin use was associated with a decreased risk of mortality in several cancers also colorectal cancer (204). However, another study showed no association with colorectal cancer incidence in United States cohort (205). Longer statin use was connected to a reduction in all-cause mortality in patients after colorectal diagnosis (206). However, overall conclusion of meta-analyses of 42 studies was that statin use was associated with a modest reduction in risk of CRC (207). This association was confirmed for lipophilic statins but not for hydrophilic statins. In addition, long-term statin use (>5 years) did not significantly affect the CRC risk.

HCC occurs mainly in cirrhotic liver and statins may affect the risk of HCC by their anti-fibrotic effect. Three meta-analyses confirmed that statin use is associated with lower risk of HCC in different populations (58, 208, 209). Fluvastatin was pointed out in one of these studies. A registry-based study also confirmed an association between statin use and the risk of HCC and additionally showed a dose response relationship (210). Stain use also significantly decreased the risk of HCC in patients with HBV (211, 212). Studies suggest that statins lower the risk of cancer in general in chronic hepatitis patients (213). Overview of studies on statin effect on mean survival rate revealed a link to extended survival, but the length of survival was variable among studies (214). Perioperative statins have been associated with improved recurrence-free survival in HCC patients (215, 216). Meta-analysis confirmed an association between statin use and decreased risk in mortality in pancreatic patients, but other studies found no association (217, 218). A review of studies revealed that observational studies found the association but randomized controlled trials did not. Statins have anti-neoplastic properties through anti-proliferative, pro-apoptotic, anti-angiogenic and immunomodulatory effects and can affect multiple signaling pathways in cells (219). Most important is the inhibition of HMGCR and by this synthesis of mevalonic acid. *HMGCR* genetic variant significantly modified the protective association between statins and CRC risk (220). Mevalonate pathway is upregulated in several cancers, also pancreatic and hepatic, and is responsible for activation of small G proteins (221). All these data make statins compelling therapy for decreasing the risk of cancer and lowering mortality of patients, but due to conflicting results, it is difficult to form final recommendations. An important issue in prescribing statins are the side effects of statins such as hepatotoxicity and drug-induced myopathy.

Since SREBPs target genes are upregulated in many cancers, SREBPs could have therapeutic potential (106). Several studies showed a successful targeting of SREBP pathway and suppression of lipid metabolism with substances like TAK1, Emodin and using different miRNA (222–225). Since oxysterols bind to INSIG and affect SREBP signaling pathway, oxysterols also have a potential to be used in cancer proliferation inhibition, but more studies on oxysterol-INSIG interaction are needed.

Oxysterols activate several nuclear receptors most importantly LXR, which is hypothesized to exert their anti-cancerogenic effects. Targeting LXR for prevention and therapy of cancers is already evaluated in clinical studies. LXR is an interesting target, because it is activated by phytosterols, which can reduce the incidence of colon cancer (226). LXR is connected to TGFB1 actions and was also proposed as a potential target for treatment of HCC (84). Bergapten, a LXR agonist, was already evaluated for HCC treatment (227). LXR agonists were proposed also for treatment of CRC since LXR activation reduced intestinal tumor formation in a mouse (APC^min/+^) CRC model and also blocked proliferation of human colorectal cells (228). LXR is a promising target, but majority of evidence was gathered through studying the effects of LXR activation in cell lines (4).

FXR is also a promising target for cancer treatment. Activation of FXR would prevent toxic bile acids build up, but also repress other tumorigenic proteins. In preclinical and clinical trials FXR agonists show a potential for treatment of different liver disease among them also HCC (175, 229, 230). In the mouse (APC^min/+^) CRC model on high fat diet, treatment with FexD, a deuterated analog of fexaramine with the gut-restricted activity, resulted in slower tumor progression, improved bile acid homeostasis and improved survival (191). However, FXR's role in pancreatic cancer is conflicting and the fact that in humans it activates a potentially pro-tumorigenic FGF19 emphasizes the needed for more studies that will confirm the positive role of FXR agonists.

### Chronotherapy as a New Therapeutic Strategy

Cancer represents the second leading cause of dead worldwide (231). The available treatment is not always the most efficient thus new therapeutic strategies are needed to be developed. In addition to standard therapy (chemotherapy, radiation, and surgery), additional factors need to be taken into consideration, such as lifestyle and biology, when providing integrative treatment for different cancer types (232). Since the circadian clock metabolism has a profound role in pathogenesis of cancers, chronotherapy might be a better therapeutic strategy. We need to adjust the pattern of drug delivery to improve the treatment efficiency, by reducing the drug at the time point where tissue is most susceptible to toxicity, and increasing the dose at times with most susceptibility to the positive drug effect (233).

Therapeutic strategy for HCC is well-established. Since the incidence is rising drastically, early diagnosis and definitive treatment is currently the only way to increase the survival rate of HCC patients. Great research breakthroughs in chronobiology led to the development of this field. Recently it was demonstrated that isoform of the HNF4A (nuclear factor 4 alpha) plays a crucial role in HCC progression. Forced expression of *BMAL1* in HCC that is positive for HNF4A stops the growth of tumors *in vivo* (234). Furthermore, SULT1A1 (sulfotransferase 1a1) that has a circadian pattern at the mRNA and protein level and is responsible for detoxifications of various drugs in the liver is regulated trough BMAL1. Knockdown of *Bmal1* resulted in changed rhythmicity in Hepa-1c1c7 cells (235). The role of IFNA (interferon alpha) was assessed in circadian manner in HepG2 cells as well. It was proposed that IFNA could have pharmacological role, since its continuous administration resulted in significantly decreased levels of CLOCK and BMAL1 protein (236).

Pancreatic cancer is one of the most aggressive tumors, responding poorly to therapy; therefore, new therapeutic strategies are in high demand. Circadian gene *Per2* overexpression increases the sensitivity to cisplatin, possibly by inducing a reduction in of the BCL (B-cell lymphoma) proteins (237). In search of cancer cell growth inhibitors downstream circadian controlled pathways, ligands of PPARG (peroxisome proliferator activated receptor gamma) showed to be reducing tumor aggressiveness and enhancing cytotoxic action of anti-cancer agents (238, 239). Another mechanism showing promise in pancreatic cancer treatment might be TIMELESS, involved in DNA damage response, whose expression was found to be altered in pancreatic cancer (135).

Chronotherapy has also proven to be effective in CRC. In metastatic CRC patients chemotherapy induced circadian disruption correlated with poorer disease outcome, suggesting its prevention could improve treatment results (240). Furthermore, a meta-analysis of five randomized controlled trials showed a significant improvement in overall survival in metastatic CRC patients treated with chronobiologically timed chemotherapy compared to conventional chemotherapy (241). Chronomodulated hepatic arterial infusion also showed promise as a possible drug administration strategy in heavily pretreated patients with CRC liver metastases (242). Recent study examining the role of PER3 in CRC found that its overexpression enhanced fluorouracil sensitivity in CRC cells, proposing it as a potential target in CRC treatment (243). Moreover, another study showed sex-specific expression and sex-specific prognostic value of clock and clock-controlled genes, shedding light on colorectal cancer and patient characteristics that have to be taken into consideration in order to provide optimal treatment (244).

## Circadian-Oxysterol Network in Cancerogenesis

Members of the oxysterol and circadian clock networks were exposed as the new promising prognostic biomarkers, genetic risk factors and potential therapeutics in gastrointestinal cancers. The roles of oxysterols in various cancers have been reviewed previously and mechanisms by which oxysterol can affect cancer pathogenesis and disease development were pointed out (8). Oxysterols activate different signaling pathways in cells which can either promote or inhibit cancer development. For example, 22(R)-, 24-, 25-, and 27-hydroxycholesterol are *in vivo* ligands of LXR and act as tumor suppressor in selected cancers. Increased LXR activity was shown to be beneficial in all three presented cancers exposing LXR as a promising drug target in oncology. However, one oxysterol can have a different role depending on the tissue. Current hypothesis is that the dual role of oxysterols is due to activation of different signaling pathways resulting in tumor suppressor LXR-dependent or oncogenic LXR-independent actions (245). Oxysterols also interact with OSBP/L proteins and these have been connected to many human diseases, such as dyslipidemia and cancers (246). Studies indicate that OSBPL family members could also have a dual role. In HCC and CRC their expression is downregulated and this is correlated with a poor prognosis. While in PC their expression is increased and correlates with a poor prognosis. The opposite roles could be explained by the fact that members of OSBPL family have tissue specific expression, which is disturbed in tumors, and also have different roles in cell physiology. Enzymes involved in oxysterol synthesis are also potential new prognostic biomarkers and drug targets in gastrointestinal cancers. The expression and the SNPs in cytochromes P450 enzymes are associated with the risk for development and prognosis of selected cancers. The level of serum cholesterol itself is a prognostic biomarker associated with a higher risk for development of numerous cancers. The association is consolidated by the fact that statins are emerging novel therapies in gastrointestinal cancers. Cholesterol is the key player not only due to being the oxysterol progenitor molecule but also one of the key cellular ingredients needed in growing cancerous cells. The oxysterol metabolites, the bile acids, and their receptor FXR are emerging new biomarkers and therapeutic targets in gastrointestinal cancers. The role of FXR in these cancers is two faced. FXR downregulation is observed with progression of cancerogenesis and FXR role in suppression of proliferation, migration and invasion in colon cells and hepatocytes was confirmed. However, FXR role in pancreatic cancer seems to be the opposite. These data indicate that FXR activity is essential in tissues with high FXR expression such as liver and colon, while FXR activity in tissues where FXR and bile acids are not common is potentially tumorigenic.

Disruptions of circadian rhythm are clearly tumorigenic in gastrointestinal cancers as indicated by rodent models. The circadian molecular clock genes are also new emerging prognostic biomarkers and therapeutic targets. Several genes involved in regulation of cellular circadian expression have direct oncogenic or tumor suppressor roles. Not only their expression correlates with prognosis in patients, knockouts in rodent models lead to spontaneous cancerogenesis and SNPs are associated with the risk of cancer development and prognosis. They are interesting therapeutic targets, moreover, the time of the therapy must also be considered and chronotherapy has already been shown to be effective in gastrointestinal cancers.

## Summary

Summary of reviewed data revealed that circadian regulatory network, cytochromes P450, nuclear receptors, cholesterol biosynthesis and oxysterols have overlapping roles in gastrointestinal cancers. Several factors from sterol homeostasis and circadian rhythm have been identified as potential novel prognostic biomarkers, genetic risk factors and drug targets. However, there are still several issues that remain open. The role of oxysterols in carcinogenesis is far from being conclusive. The cells are dealing with a variety of oxysterol molecules that promote their actions through modulation of transcription factors, such as LXR, RORs and others. Depending on the type of cancer, an oxysterol modulated pathway can have beneficial or damaging effect on carcinogenesis. This is one of the obstacles in developing new therapeutic strategies, besides statins, where it is clear that a more personalized approach is essential to increase the positive drug effects. The link between cholesterol, oxysterol synthesis and circadian rhythm was until recently mostly unidirectional: the clock controlled the expression of lipogenic genes. It now becomes evident that both pathways are interconnected by energy metabolism where RORA/C and LXR are at the crossroad. The ROR receptors are circadian regulators, being co-responsible for the rhythmic expression of output metabolic genes. However, their own transcriptional activity depends on sterols and oxysterols whose endogenous synthesis is regulated by the clock. This is again a new field of translational research termed chronotherapy which was proven to be successful particularly in treatments of colorectal cancer.

## Author Contributions

All authors listed have made a substantial, direct and intellectual contribution to the work, and approved it for publication.

### Conflict of Interest Statement

The authors declare that the research was conducted in the absence of any commercial or financial relationships that could be construed as a potential conflict of interest.
